# Neighborhood Light at Night and Noise Levels, and Long-Term Sleep Trajectories in the Southern Community Cohort Study

**DOI:** 10.3390/clockssleep6020016

**Published:** 2024-04-05

**Authors:** Samuel H. Nyarko, Qian Xiao

**Affiliations:** 1 Department of Epidemiology, Human Genetics and Environmental Sciences, School of Public Health, The University of Texas Health Science Center at Houston (UTHealth), Houston, TX 77030, USA; qian.xiao@uth.tmc.edu; 2 Department of Epidemiology and Biostatistics, Arnold School of Public Health, University of South Carolina, Columbia, SC 29208, USA

**Keywords:** noise, sleep duration, short sleep, normal sleep, long sleep

## Abstract

While light at night (LAN) and noise levels have been linked to suboptimal sleep outcomes, little is known about the link between these factors and long-term suboptimal sleep trajectories. The current study examined the association of neighborhood LAN and nighttime noise with long-term sleep trajectories in a cohort of Black individuals and White individuals predominantly from low-income communities. We used data from the Southern Community Cohort Study (N = 28,759 Black individuals and 16,276 White individuals). Sleep duration was self-reported at baseline and after an average of five years of follow-up, based on which we constructed nine sleep trajectories: normal–normal (optimal, reference), short–short, long–long, short–long, long–short, normal–short, normal–long, short–normal, and long–normal. LAN and nighttime noise were derived from satellite imagery and model-based estimates, respectively. Multinomial logistic regression was used to determine the relationship between LAN and noise exposures and sleep trajectories. Higher exposures to LAN and nighttime noise were associated with multiple suboptimal long-term sleep trajectories. In the total sample, higher LAN was associated with higher odds of long–long (OR _Q5 vs. Q1_ = 1.23 (CI = 1.02, 1.48)) and long–short (OR = 1.35 (CI = 1.06, 1.72)) trajectories, while higher nighttime noise was associated with short–short (1.19 (1.07, 1.31)), long–short (1.31 (1.05, 1.64)), and normal–song (1.16 (1.01, 1.34)) trajectories. Black and White individual-specific results showed qualitatively similar patterns between Black individuals and White individuals, although we also observed suggestive evidence for Black–White individual differences. In conclusion, elevated LAN and nighttime noise levels were associated with various suboptimal long-term sleep trajectories. However, it is noteworthy that the light and noise measures in our study may not accurately reflect individual-level exposures, and residual confounding from other factors is a concern. Future studies should use more accurate exposure measurements, collect information on and control for a wider range of factors, and examine whether reductions in neighborhood light and noise levels may contribute to improved long-term sleep health.

## 1. Introduction

Healthy sleep is vital to health and well-being [1]. It is recommended that adults regularly take 7 to 9 h of sleep per day to support optimal health [2,3]. Both short and long sleep durations have been linked to increased risks for various adverse health outcomes, including all-cause mortality [4,5,6], obesity, diabetes, hypertension, and cardiovascular disease [7]. Moreover, sleep is a dynamic behavior that may change over time. More recently, several studies suggested that changes in sleep and long-term sleep trajectories are unique predictors of health concerns and diseases, such as hypertension [8,9], systemic inflammation [10,11], cardiovascular events, and all-cause mortality [12,13]. Considering the important health implications of sleep patterns, it is important to study the factors that may shape sleep behavior and contribute to health outcomes.

Sleep is not only determined by internal and individual-level factors but also influenced by environmental exposures. In particular, a burgeoning body of research has linked high levels of environmental light and noise exposure at night to suboptimal sleep outcomes [14,15,16,17,18]. The endogenous circadian clock has evolved as a fundamental adaptation to the 24 h light and dark cycle on Earth, and daily fluctuation in ambient light levels plays a critical role in maintaining rhythmic behaviors and physiological processes under circadian control. Pervasive exposure to light at night (LAN) suppresses melatonin, a key hormone in circadian regulation, and enables nighttime activities that are misaligned with the internal circadian clock. Both melatonin suppression and misaligned nighttime activities can lead to circadian disruption and sleep deficiencies [19,20]. Indeed, epidemiological studies have linked higher levels of outdoor LAN with delayed bedtime and shorter sleep duration in various populations [14,15,16]. Similarly, exposure to environmental noise at night, primarily from traffic noise (e.g., road, rail, and aircraft), has also been shown to be a major contributor to sleep disturbances and daytime sleepiness [17,21]. The World Health Organization estimated that in the European Union and other western European countries, noise-related sleep problems contributed to over 900 thousand disability-adjusted life years lost every year [22]. Moreover, decades of environmental justice research have documented a disproportionately high level of environmental hazardous exposures in disadvantaged neighborhoods, including LAN and noise [23,24]. However, to the best of our knowledge, no study has investigated the potential impact of neighborhood levels of light and noise at night on sleep health in low-income and minoritized populations, which has limited our understanding of the impact of environmental exposures on long-term sleep health. Studying and understanding environmental determinants of long-term sleep trajectories helps to identify high-risk populations, uncover contributors to adverse health outcomes, and guide interventions [5,12,25].

To address these gaps, the current study examined the association of neighborhood LAN and nighttime noise levels with long-term sleep trajectories among Black individuals and White individuals in the Southern Community Cohort Study, a cohort that enrolled participants from predominantly low-income communities. We hypothesize that higher levels of LAN and nighttime noise in the neighborhood are associated with suboptimal sleep trajectories in both Black and White individuals.

## 2. Results

Table 1 presents descriptive statistics of selected baseline characteristics by the nine long-term sleep trajectories in the total sample, and Black individuals and White individuals specifically. In the total sample, when compared to the stable healthy group, individuals with a suboptimal sleep trajectory were younger, more likely to be female, report household income < USD 15,000, and live in the lowest quintile of neighborhood SES, but were less likely to have a college education. Both LAN and nighttime noise levels were higher among individuals with a suboptimal sleep trajectory compared with those in the stable healthy group. These patterns were largely similar among Black and White individuals. Finally, when compared to White individuals, Black individuals were younger and less likely to have a college degree or higher, but were more likely to report household income < USD 15,000 and live in the lowest quintile of neighborhood SES. LAN and nighttime noise levels were higher among Black individuals than White individuals.

The associations between LAN and all nine sleep trajectories are presented in Table 2. In the total sample, LAN was positively and significantly associated with the long–long and long–short trajectories (OR _Q5 vs. Q1_, (95% CI), 1.23 (1.02, 1.48) and 1.35 (1.06, 1.72), respectively). There was also a suggestive positive association with the normal–short trajectory, with borderline statistical significance (1.14 (0.99, 1.31). Black and White individual-specific results were qualitatively similar; however, we also noticed potential differences between Black and White individual subsamples. For example, there was a significant association between higher LAN and the short–short and normal–short trajectories in Black individuals (1.16 (1.00, 1.32) and 1.20 (1.01, 1.42), respectively), but not White individuals (1.06 (0.87, 1.29) and 1.06 (0.81, 1.39)). In contrast, higher LAN was associated with the normal–long trajectory in White individuals (1.47 (1.14, 1.90)), but not Black individuals (0.94 (0.77, 1.15)).

Table 3 presents associations between neighborhood nighttime noise levels and nine-category long-term sleep trajectories. In the total sample, higher noise levels were associated with higher odds of the short–short (1.19 (1.07, 1.31)), long–short (1.31 (1.05, 1.64)), and normal–long (1.16 (1.01, 1.34)) trajectories. In Black and White individual-specific analysis, the associations appeared stronger for multiple unfavorable sleep trajectories among Black individuals than among White individuals (short–short (Black individuals: 1.25 (1.10, 1.43), White individuals: 1.10 (0.92, 1.32)), long–short (1.39 (1.08, 1.79), 1.07 (0.60, 1.90)), and normal–short (1.22 (1.04, 1.42), 0.81 (0.63, 1.04)), as well as the favorable sleep trajectories (short–normal (1.15 (1.00, 1.33), 0.98 (0.80, 1.19)) and long–normal (1.21 (1.00, 1.46), 1.11 (0.82, 1.51)). However, the relationship between noise and the normal–long trajectory appeared stronger in White individuals (1.35 (1.06, 1.71)) than in Black individuals (1.07 (0.89, 1.28)).

In secondary analysis, which focused on the association between light and noise exposure and sleep duration at baseline and follow-up, separately, we found that the highest quintiles of LAN and nighttime noise levels were statistically associated with higher odds of long sleep at the baseline and short sleep at the follow-up among the total sample. There were Black–White individual disparities similar to the observed disparities in the main analysis (Supplementary Tables S1 and S2).

## 3. Discussion

This study examined the association between neighborhood LAN and nighttime noise levels and long-term sleep trajectories within a large prospective cohort of middle-to-older-aged Black individuals and White individuals recruited from low-income communities in the Southeastern United States. The findings mainly showed that residents of neighborhoods with higher LAN and nighttime noise levels were more likely to experience suboptimal sleep trajectories over time, and the results were similar in both Black and White individuals, although we also observed suggestive Black–White individual differences.

A growing body of research has linked LAN exposure to various suboptimal sleep outcomes [14,16,18,26,27,28,29,30]. For example, in a Japanese cohort of older adults, higher levels of bedroom light were associated with a shortened sleep duration and poor sleep quality [29,30]. Moreover, using satellite-based LAN estimates, Xiao and colleagues found that higher neighborhood LAN levels were associated with higher odds of both short and long sleep durations in the NIH-AARP Diet and Health Study in the United States [16]. Similarly, in a cross-sectional study of a representative sample of the US population, higher levels of satellite-based LAN measures were associated with more wakening during sleep, increased daytime sleepiness, and short sleep duration [15]. Furthermore, multiple studies have suggested that environmental noise stemming from traffic and other anthropogenic noise may have an adverse effect on sleep duration and quality. For example, higher levels of neighborhood noise have been linked to sleep disturbances, short sleep duration, insomnia, and daytime sleepiness among human populations [17,21,31,32,33,34,35,36]. Similarly, the growing body of literature has shown that noise pollution from neighborhood aircraft, rail, and roadways makes it challenging to fall and stay asleep, which may lead to sleep disturbances [26,31]. However, all the aforementioned studies were cross-sectional and focused on sleep duration measured at a single time point. In contrast, our study used repeated measures of sleep duration to construct multiple long-term sleep trajectories, and the findings suggest that baseline exposure to neighborhood LAN and nighttime noise are important predictors of long-term sleep patterns.

A growing number of studies have shown that long-term sleep trajectories are unique predictors of health outcomes. For instance, consistent short and long sleep duration over time has been linked to multiple adverse health effects on human health, including systemic inflammation, type 2 diabetes, hypertension, and mortality [8,9,10,11,12,13]. Moreover, unstable or fluctuating sleep duration over time has been linked to increased risk of cardiovascular diseases and all-cause mortality [12,13,37], as well as elevated levels of inflammatory markers, such as C-reactive protein and interleukin-6 [10,11], which can contribute to many diseases, including coronary heart disease, stroke, and all-cause mortality [38,39]. Furthermore, individuals with either substantial changes in sleep duration or persistently short sleep over time have been shown to have an increased risk of hypertension and higher levels of its biomarkers, such as uric acid, fasting glucose, and total cholesterol [8,9]. Finally, it has been shown that those whose sleep duration increased consistently over time had a higher risk of type 2 diabetes [40].

Our results suggested that higher levels of neighborhood LAN and nighttime noise were associated with suboptimal sleep trajectories in both Black and White individuals. It is worth noting that there exist persistent Black–White individual disparities in exposure to environmental hazards in the US. In particular, a growing body of research has found that neighborhoods with a high percentage of Black residents have higher levels of noise and light pollution than predominantly White neighborhoods. For instance, a study conducted by Casey et al. found that nighttime noise levels in urban block groups with 75% Black residents were 46.3 decibels, compared with 42.3 decibels for block groups with 0% Black residents [23]. Similarly, another study reported that the mean exposure to neighborhood LAN of Black American individuals was two times that of White American individuals [24]. Our own analyses using the overall and domain-specific measures of social vulnerability also revealed a substantially higher exposure level of LAN in more disadvantaged populations, particularly minoritized populations [41]. Additionally, multiple studies have shown that Black individuals are more likely to experience suboptimal sleep trajectories than White individuals [42,43,44,45]. Evidence also suggests that when compared with White individuals, Black individuals have higher burdens of diseases related to suboptimal sleep patterns, including diabetes [46], hypertension [47,48], obesity [49], and cardiometabolic diseases [50]. The subgroup analysis conducted separately in Black and White individuals is crucial for understanding unique factors contributing to suboptimal sleep patterns in these populations. Future studies should examine the contribution of uneven distribution of LAN and nighttime noise to disparities in sleep health and other sleep-related health outcomes (e.g., cardiovascular disease, obesity, diabetes, and dementia) in the population. This will be essential in providing a contextual understanding about the root causes of disparities to ensure the design of effective interventions for reducing long-term sleep disparities based on neighborhood LAN and noise characteristics.

This study has several limitations. First, sleep duration was measured based on self-reported information. Future studies using objective measurements of sleep are needed to provide a more accurate sleep assessment. Second, sleep is a multidimensional behavior [51], but the present study only analyzed sleep duration and lacked measures of other dimensions of sleep. Consequently, future studies are needed to investigate other dimensions of sleep. Third, LAN was based on a satellite measurement that represents outdoor lighting levels and may not necessarily reflect actual light exposure levels experienced by people, particularly in an indoor environment. Similarly, the model-derived noise level may also deviate from the indoor noise exposure experienced by the individual. For example, the actual exposure levels to light and noise can be impacted by many factors, including window treatment, indoor lighting conditions, the floor level of the bedroom, facing directions of the room, sleep hygiene practices, domestic obligations (e.g., childcare and senior care), and work schedule (e.g., shift work). However, we did not have measures of these factors and were not able to investigate their impact on our results in this analysis. Fourth, noise was also not based on actual measurements, but model-derived measurements, and thus could be inaccurate. Fifth, although we adjusted for many sociodemographic characteristics of participants, including rural–urban residential status and the neighborhood deprivation index, there could still be residual confounding due to other environmental exposures that correlate with LAN and noise and have an impact on sleep. Lastly, the study sample comprised only Black and White individuals, and thus the results cannot be generalized to other groups. Nevertheless, our study has several unique strengths. First, to the best of our knowledge, ours is the first study to examine neighborhood LAN and nighttime noise in relation to long-term sleep patterns. Second, the SCCS is a large cohort with diverse populations, which allowed us to conduct Black–White individual-specific analysis. Third, we focused on predominantly low-income populations, who have a high burden of hazardous exposures but are often overlooked in previous studies.

## 4. Methods

### 4.1. Data and Study Population

We used data from the Southern Community Cohort Study (SCCS) to conduct the analysis. The SCCS is a prospective study comprising middle-to-older-aged adults (40–79 years) from 12 states (Alabama, Arkansas, Florida, Georgia, Kentucky, Louisiana, Mississippi, North Carolina, South Carolina, Tennessee, Virginia, and West Virginia) in the southeastern United States. At baseline (2002–2009), ~80% of the study sample were recruited from community health centers serving underinsured and uninsured populations and had relatively low socioeconomic status (SES). About two-thirds of the baseline cohort were Black individuals. The SCCS enrolled 84,508 individuals during the baseline survey and obtained information on residential address, sociodemographic and behavioral characteristics, as well as health status. In 2008–2013, a follow-up survey was mailed to the baseline cohort and collected updated information on many study variables, including sleep duration. Details of the survey design and study population have been documented by Signorello et al. [52]. The Institutional Review Boards of Vanderbilt University and Meharry Medical College approved the study protocols of the SCCS. Study participants provided informed consent before inclusion in the study. Within the initial cohort, 75,248 Black and White individuals reported residential addresses during the baseline survey. We excluded 30,213 individuals who had missing sleep duration data at the baseline and/or the follow-up survey, including those who moved between the baseline and follow-up periods. The final analytical sample consisted of 45,035 individuals (N = 28,759 Black individuals and 16,276 White individuals). 

### 4.2. Sleep Trajectories

During both baseline and follow-up surveys, participants reported total hours of sleep in a 24 h period on weekdays and weekends, separately. Both baseline and follow-up sleep durations were calculated as the weighted average of the number of sleep hours on weekdays and weekends (5 × weekday duration + 2 × weekend duration/7) and were then categorized into short (<7 h), normal (7-<9 h), and long (≥9 h) sleep durations. We then derived the primary outcome of the study comprising nine sleep duration trajectories between the baseline and follow-up periods: normal–normal, short–short, long–long, short–long, long–short, normal–short, normal–long, short–normal, and long–normal. The normal–normal category was considered to present the optimal sleep pattern and was used as the reference group. Sleep duration categories (i.e., short, normal (reference), and long) at baseline and follow-up, separately, were also used as a secondary outcome to perform sensitivity analysis.

### 4.3. Light and Noise Measurements

The exposure variables were LAN and nighttime noise levels, which were linked to the residential addresses of the participants. The LAN was obtained from satellite imagery data for 2006 from the United States Air Force Defense Meteorological Satellite Program’s Operational Linescan System (DMSP-OLS) [53]. We used the DMSP Global Radiance Calibrated Nighttime Lights high-dynamic range data, which captured a larger dynamic range of nighttime illumination and can be transformed into units of radiance (nanowatts/cm^2^/sterradian(sr)) [54]. We used the annual composites measure, which was derived after excluding sun and moon luminance, glare, clouds, atmospheric lighting, and ephemeral events, such as fires, and had a spatial resolution at a 30 arc-second grid (equivalent to ~1 km-sq). The noise level was estimated using a geospatial model of environmental sound levels developed by the National Park Service, providing data at a resolution of 270 m [55]. We used the median level of anthropogenic noise (dBA) at night (period between 10 p.m. and 7 a.m.) as the noise exposure variable in this analysis. The values of the LAN and nighttime noise variables were extracted from raster data and merged with the SCCS using geographic coordinates of the baseline residential addresses of individuals in the sample. The continuous values of the two outcomes were then divided into quintiles (Q1 (lowest, reference), Q2, Q3, Q4, and Q5 (highest)).

### 4.4. Covariates

We included the following baseline variables as covariates in this analysis: age (40–49, 50–59, 60–69, and 70–79), sex (male/female), race/ethnicity (Black and White individuals), educational attainment (<high school, high school, vocational/some college, and college/higher), household income (USD: <15,000, 15,000–24,999, 25,000–49,999, and 50,000+), metropolitan status of residence (metro or non-metro), and neighborhood socioeconomic status (SES) index (quintiles). Age, sex, race/ethnicity, education, and income were based on self-reported information. The metropolitan residential status of participants was categorized as metro or non-metro based on the 2003 Rural–Urban Continuum Codes. Neighborhood SES was measured using a deprivation index developed by Messer et al. [56]. Fourteen census tract-level variables (percent of total less than high school, percent of total unemployed, percent of households with income below poverty, percent of households with an income < USD 30,000, percent of households on public assistance, percent of households with no car, percent of unemployed men, percent of renter-occupied housing units, percent of housing units vacant, median value of all owner-occupied housing units, percent of female-headed household with dependent children, percent of non-Hispanic Black individuals, percent of residents 65 years and above, and percent of persons in the same residence since 5 years before the census) were used in a principal component analysis to calculate the index, which was then divided into quintiles and reverse-coded to indicate the highest and lowest SES.

### 4.5. Statistical Analysis

Descriptive statistics of background characteristics were presented in the forms of means, standard deviation, interquartile range, and percentages for each of the five sleep trajectory groups and for both the total sample and Black and White individuals separately. Pearson’s Chi-squared and Kruskal–Wallis’ rank sum tests were used to test differences in baseline characteristics across different sleep trajectory groups. Multinomial logistic regression models were used to examine the association between LAN and nighttime noise and long-term sleep trajectories, using the normal–normal trajectory (stable, healthy) as the reference category. Models were adjusted for age, sex, race/ethnicity (total sample alone), education, household income, metropolitan status of residence, and deprivation index. We performed regression analysis in the total sample and for Black and White individuals separately. For a secondary analysis, the analyses were repeated for sleep duration at both the baseline and follow-up time points. Interaction analyses were conducted by including a product term between Black and White individuals and LAN, as well as nighttime noise in the main model. All analyses were performed with the R programming language (version 4.0.3) [57].

## 5. Conclusions

We found that higher neighborhood LAN and nighttime noise levels were prospectively associated with suboptimal long-term sleep trajectories, particularly among Black individuals in a predominantly low-income population. Considering the growing evidence suggesting a high burden of LAN and nighttime noise exposure in Black individuals’ neighborhoods and well-documented Black–White individual disparities in sleep health and other sleep-related health outcomes, it is a priority for future public health research to evaluate the contribution of emerging environmental hazards, such as light and noise pollution, to health disparities. We also encourage future studies to employ study designs (e.g., randomized intervention studies) and analytic strategies (e.g., causal modeling) that can help elucidate a potential cause-and-effect relationship between environmental exposures and sleep patterns. Findings from such studies can inform public health interventions aiming at mitigating environmental hazards in minoritized neighborhoods to improve long-term sleep health and reduce disease burden in vulnerable populations.

## Figures and Tables

**Table 1:** Baseline characteristics by long-term sleep trajectories among Black and White individuals.

Characteristic	Normal–Normal	Short–Short	Long–Long	Short–Long	Long–Short	Normal–Short	Normal–Long	Short–Normal	Long–Normal
**Black individuals**									
N (%)	6951 (24.2)	6095 (21.2)	1377 (4.8)	1519 (5.3)	979 (3.4)	3205 (11.1)	2111 (7.3)	4547 (15.8)	1975 (6.9)
Age, Mean (SD)	53 (8.7)	51 (7.9)	52 (8.8)	52 (8.3)	51 (8.4)	52 (8.6)	53 (9.0)	52 (8.2)	52 (8.5)
Female, %	65.9	68.4	66.0	63.8	65.6	67.1	63.3	66.4	63.2
College/higher, %	15.7	15.6	9.0	8.4	8.2	11.7	10.6	13.9	9.0
Household income, <USD 15,000, %	49.4	49.8	63.0	62.7	64.7	55.3	58.7	51.0	59.3
Metro residence, %	75.5	79.5	77.0	77.0	76.9	75.7	75.5	77.1	74.3
Neighborhood SES (lowest), %	55.7	54.7	62.5	61.6	61.6	57.1	60.1	56.8	61.4
LAN, highest quintile, %	19.7	20.5	22.3	22.1	24.5	21.8	20.0	19.8	22.8
LAN, median (IQR)	172.9 (239.8)	191.2 (228.6)	197.6 (236.3)	185.8 (245.0)	198.7 (249.6)	185.8 (243.3)	191.2 (244.9)	186.6 (227.6)	191.7 (253.4)
Noise, highest quintile, %	19.5	21.2	22.9	20.9	25.0	22.0	20.8	20.1	21.6
Noise, median (IQR)	45.1 (4.3)	45.6 (4.2)	45.7 (4.2)	45.7 (4.2)	46.0 (4.3)	45.4 (4.4)	45.5 (4.3)	45.5 (4.2)	45.5 (4.3)
**White individuals**									
N (%)	5938 (36.5)	3012 (18.5)	751 (4.6)	679 (4.2)	187 (1.1)	1282 (7.9)	1303 (8.0)	2400 (14.7)	724 (4.4)
Age, Mean (SD)	56 (9.1)	53 (8.5)	56 (9.1)	53 (8.9)	52 (8.6)	54 (9.2)	56 (9.2)	55 (8.8)	55 (8.9)
Female, %	60.6	67.6	62.1	71.0	65.2	68.3	65.0	65.4	66.0
College/higher, %	34.4	17.3	21.4	8.5	9.1	20.6	23.0	20.5	18.0
Household income, <USD 15,000, %	26.8	46.9	46.8	61.4	58.5	40.3	43.7	40.7	48.5
Metro residence, %	74.3	78.5	75.6	76.4	76.5	74.1	76.0	74.8	74.4
Neighborhood SES (lowest), %	9.9	13.9	13.4	16.4	16.9	14.6	14.3	11.4	15.2
LAN, highest quintile, %	6.9	9.4	10.4	9.6	13.4	9.1	11.1	7.4	10.9
LAN, median (IQR)	62.3 (152.1)	66.5 (169.3)	67.3 (168.7)	63.3 (178.8)	65.9 (180.2)	55.7 (159.4)	75.6 (179.9)	59.8 (151.5)	67.1 (175.8)
Noise, highest quintile, %	7.6	10.5	11.5	10.3	10.7	8.5	11.3	8.7	10.7
Noise, median (IQR)	43.5 (3.8)	43.7 (4.6)	43.6 (4.1)	43.6 (4.9)	43.7 (5.3)	43.5 (4.6)	43.7 (4.5)	43.5 (4.2)	43.8 (4.7)
**Total sample**									
N (%)	12,889 (28.6)	9107 (20.2)	2128 (4.7)	2198 (4.9)	1166 (2.6)	4487 (10.0)	3414 (7.6)	6947 (15.4)	2699 (6.0)
Age, Mean (SD)	54 (9.1)	52 (8.2)	53 (9.1)	52 (8.5)	51 (8.5)	53 (8.8)	54 (9.2)	53 (8.5)	53 (8.7)
Female, %	63.4	68.1	64.6	66.0	65.5	67.4	63.9	66.1	64.0
College/higher, %	24.3	16.1	13.4	8.4	8.3	14.2	15.3	16.2	11.4
Household income, <USD 15,000, %	39.0	48.9	57.3	62.3	63.7	51.0	53.0	47.5	56.4
Metro residence, %	75.0	79.2	76.5	76.8	76.8	75.2	75.7	76.3	74.4
Neighborhood SES (lowest), %	34.8	41.3	45.3	47.7	54.6	45.1	42.9	41.3	49.1
LAN, highest quintile, %	13.8	16.8	18.2	18.2	22.8	18.2	16.6	15.5	19.6
LAN, median (IQR)	114.5 (220.5)	145.7 (232.6)	146.9 (241.4)	143.8 (248.1)	170.2 (256.0)	143.8 (243.1)	143.8 (242.4)	133.9 (231.5)	152.2 (255.9)
Noise, highest quintile, %	14.0	17.7	18.9	17.7	22.7	18.2	17.1	16.2	18.6
Noise, median (IQR)	44.3 (4.5)	44.8 (4.6)	44.8 (4.6)	44.8 (4.6)	45.5 (4.4)	44.7 (4.6)	44.7 (4.6)	44.6 (4.5)	44.9 (4.5)

IQR, interquartile range; SD, standard deviation; SES, socioeconomic status; LAN, light at night. All Kruskal–Wallis rank sum and Pearson’s Chi-squared test *p*-values were significant at <0.001.

**Table 2:** Multinomial regression analysis of neighborhood LAN levels and long-term trajectories.

	Normal–Normal	Short–Short	Long–Long	Short–Long	Long–Short	Normal–Short	Normal–Long	Short–Normal	Long–Normal
**LAN Levels**		OR	OR	OR	OR	OR	OR	OR	OR
**Black individuals**									
Q1 (Lowest)	Ref	Ref	Ref	Ref	Ref	Ref	Ref	Ref	Ref
Q2	Ref	1.05 (0.93, 1.19)	1.13 (0.91, 1.39)	0.99 (0.81, 1.21)	1.26 (0.99, 1.61)	1.00 (0.87, 1.16)	0.81 (0.68, 0.96)	0.94 (0.83, 1.07)	0.99 (0.83, 1.18)
Q3	Ref	1.16 (1.02, 1.31)	1.30 (1.05, 1.62)	1.05 (0.86, 1.29)	1.13 (0.87, 1.47)	1.08 (0.92, 1.25)	0.94 (0.79, 1.13)	1.02 (0.89, 1.17)	1.02 (0.85, 1.23)
Q4	Ref	1.21 (1.06, 1.37)	1.22 (0.97, 1.52)	0.96 (0.78, 1.19)	1.12 (0.86, 1.47)	1.05 (0.90, 1.23)	1.08 (0.90, 1.29)	1.07 (0.93, 1.24)	1.06 (0.87, 1.28)
Q5 (Highest)		1.16 (1.00, 1.33)	1.24 (0.97, 1.57)	0.99 (0.79, 1.24)	1.33 (1.01, 1.75)	1.20(1.01, 1.42)	0.94 (0.77, 1.15)	0.99 (0.85, 1.15)	1.15 (0.94, 1.41)
**White individuals**									
Q1 (Lowest)	Ref	Ref	Ref	Ref	Ref	Ref	Ref	Ref	Ref
Q2	Ref	1.06 (0.94, 1.20)	1.16 (0.94, 1.44)	0.95 (0.76, 1.19)	1.21 (0.80, 1.84)	0.95 (0.80, 1.12)	0.98 (0.82, 1.16)	1.04 (0.91, 1.18)	1.02 (0.83, 1.27)
Q3	Ref	1.01 (0.87, 1.16)	1.26 (0.98, 1.61)	1.00 (0.77, 1.29)	1.14 (0.70, 1.84)	0.99 (0.81, 1.20)	1.12 (0.93, 1.37)	1.08 (0.93, 1.26)	0.97 (0.75, 1.25)
Q4	Ref	1.01 (0.85, 1.20)	0.97 (0.71, 1.32)	1.04 (0.77, 1.41)	0.96 (0.53, 1.74)	0.94 (0.74, 1.20)	1.08 (0.85, 1.36)	0.94 (0.77, 1.13)	0.95 (0.70, 1.29)
Q5 (Highest)	Ref	1.06 (0.87, 1.29)	1.36 (0.98, 1.89)	0.97 (0.68, 1.37)	1.51 (0.84, 2.74)	1.06 (0.81, 1.39)	1.47 (1.14, 1.90)	0.95 (0.76, 1.19)	1.29 (0.93, 1.79)
**Total sample**									
Q1 (Lowest)	Ref	Ref	Ref	Ref	Ref	Ref	Ref	Ref	Ref
Q2	Ref	1.06 (0.97, 1.15)	1.15 (0.99, 1.34)	0.96 (0.83, 1.11)	1.29 (1.05, 1.58)	0.99 (0.88, 1.10)	0.91 (0.80, 1.02)	0.98 (0.89, 1.07)	1.00 (0.87, 1.14)
Q3	Ref	1.09 (0.99, 1.19)	1.28 (1.09, 1.50)	1.01 (0.86, 1.18)	1.17 (0.93, 1.46)	1.04 (0.92, 1.17)	1.02 (0.90, 1.16)	1.02 (0.92, 1.13)	0.98 (0.85, 1.14)
Q4	Ref	1.10 (1.00, 1.22)	1.14 (0.95, 1.36)	0.93 (0.79, 1.10)	1.11 (0.87, 1.40)	0.99 (0.87, 1.13)	1.12 (0.98, 1.29)	1.01 (0.90, 1.12)	1.00 (0.86, 1.17)
Q5 (Highest)	Ref	1.07 (0.96, 1.20)	1.23 (1.02, 1.48)	0.94 (0.79, 1.13)	1.35 (1.06, 1.72)	1.14 (0.99, 1.31)	1.09 (0.94, 1.27)	0.94 (0.84, 1.06)	1.13 (0.95, 1.33)

Models were adjusted for race/ethnicity (total sample alone), age, sex, education, household income, metropolitan status of residence, and deprivation index. Ref=Reference category; OR = Odds Ratio.

**Table 3:** Multinomial regression analysis of neighborhood noise levels and long-term trajectories.

	Normal–Normal	Short–Short	Long–Long	Short–Long	Long–Short	Normal–Short	Normal–Long	Short–Normal	Long–Normal
**Noise levels**		OR	OR	OR	OR	OR	OR	OR	OR
**Black individuals**									
Q1 (Lowest)	Ref	Ref	Ref	Ref	Ref	Ref	Ref	Ref	Ref
Q2	Ref	1.02 (0.90, 1.15)	1.07 (0.87, 1.31)	1.01 (0.84, 1.23)	1.05 (0.82, 1.34)	0.93 (0.81, 1.07)	0.96 (0.81, 1.13)	1.09 (0.96, 1.24)	1.12 (0.94, 1.34)
Q3	Ref	1.13 (1.00, 1.27)	1.14 (0.93, 1.40)	1.05 (0.87, 1.28)	1.10 (0.86, 1.40)	1.05 (0.91, 1.22)	1.06 (0.89, 1.25)	1.16 (1.02, 1.32)	1.20 (1.01, 1.43)
Q4	Ref	1.25 (1.11, 1.42)	1.11 (0.90, 1.37)	1.13 (0.92, 1.37)	1.22 (0.96, 1.56)	1.09 (0.94, 1.27)	1.12 (0.94, 1.33)	1.21 (1.05, 1.38)	1.16 (0.96, 1.39)
Q5 (Highest)		1.25 (1.10, 1.43)	1.20 (0.96, 1.49)	1.02 (0.83, 1.26)	1.39 (1.08, 1.79)	1.22 (1.04, 1.42)	1.07 (0.89, 1.28)	1.15 (1.00, 1.33)	1.21 (1.00, 1.46)
**White individuals**									
Q1 (Lowest)	Ref	Ref	Ref	Ref	Ref	Ref	Ref	Ref	Ref
Q2	Ref	1.06 (0.93, 1.20)	1.04 (0.84, 1.29)	0.97 (0.78, 1.22)	1.27 (0.85, 1.91)	0.92 (0.78, 1.08)	1.22 (1.03, 1.44)	1.03 (0.90, 1.18)	0.93 (0.75, 1.16)
Q3	Ref	0.98 (0.85, 1.13)	1.16 (0.92, 1.46)	0.86 (0.66, 1.11)	0.97 (0.60, 1.55)	0.81 (0.67, 0.97)	1.06 (0.87, 1.27)	0.94 (0.82, 1.09)	1.02 (0.80, 1.29)
Q4	Ref	1.10 (0.94, 1.28)	0.91 (0.69, 1.20)	1.04 (0.79, 1.35)	1.24 (0.77, 1.99)	0.88 (0.71, 1.08)	1.16 (0.94, 1.43)	1.05 (0.89, 1.24)	1.10 (0.85, 1.43)
Q5 (Highest)	Ref	1.10 (0.92, 1.32)	1.22 (0.90, 1.63)	0.90 (0.66, 1.24)	1.07 (0.60, 1.90)	0.81 (0.63, 1.04)	1.35 (1.06, 1.71)	0.98 (0.80, 1.19)	1.11 (0.82, 1.51)
**Total sample**									
Q1 (Lowest)	Ref	Ref	Ref	Ref	Ref	Ref	Ref	Ref	Ref
Q2	Ref	1.04 (0.95, 1.13)	1.04 (0.90, 1.21)	0.99 (0.86, 1.14)	1.08 (0.88, 1.32)	0.91 (0.82, 1.01)	1.08 (0.96, 1.22)	1.05 (0.96, 1.15)	1.05 (0.92, 1.20)
Q3	Ref	1.07 (0.98, 1.17)	1.11 (0.95, 1.28)	0.99 (0.85, 1.15)	1.03 (0.84, 1.28)	0.94 (0.84, 1.05)	1.09 (0.96, 1.23)	1.05 (0.95, 1.15)	1.11 (0.97, 1.27)
Q4	Ref	1.21 (1.10, 1.32)	1.03 (0.87, 1.21)	1.10 (0.95, 1.29)	1.19 (0.96, 1.48)	1.00 (0.89, 1.12)	1.15 (1.01, 1.32)	1.12 (1.01, 1.24)	1.12 (0.97, 1.30)
Q5 (Highest)	Ref	1.19 (1.07, 1.31)	1.15 (0.97, 1.37)	0.97 (0.82, 1.15)	1.31 (1.05, 1.64)	1.07 (0.94, 1.21)	1.16 (1.01, 1.34)	1.06 (0.95, 1.18)	1.14 (0.98, 1.33)

Models were adjusted for race/ethnicity (total sample alone), age, sex, education, household income, metropolitan status of residence, and deprivation index. Ref=Reference category; OR = Odds Ratio.

## Data Availability

The data analyzed in the current study are available from: https://www.southerncommunitystudy.org, upon request (accessed on 8 September 2020).

## Supplementary Materials

The following supporting information can be downloaded at: https://www.mdpi.com/article/10.3390/clockssleep6020016/s1, Supplementary Table S1: Multinomial regression analysis of neighborhood LAN levels and sleep durations at baseline and follow-up; Supplementary Table S2: Multinomial regression analysis of neighborhood noise levels and sleep durations at baseline and follow-up.
