# Supplementary material for: Neighborhood Light at Night and Noise Levels, and Long-Term Sleep Trajectories in the Southern Community Cohort Study
Source: Clocks Sleep. 2024 Apr 5;6(2):234–45. doi: 10.3390/clockssleep6020016 (PMC11036205; doi:10.3390/clockssleep6020016)
Supplement: Supplementary file 1 [file clockssleep-06-00016-s001.zip › clockssleep-2841944-supplementary.pdf]

Supplementary Table 1: Multinomial regression analysis of neighborhood LAN levels and sleep durations at baseline and follow-up

	Baseline		Follow-up	
Characteristic	Short sleep	Long sleep	Short sleep	Long sleep
	OR	OR	OR	OR
**LAN**		**BLACK**		
Q1(Lowest)	Ref	Ref	Ref	Ref
Q2	1.03 (0.94, 1.13)	1.12 (0.99, 1.27)	1.07 (0.98, 1.18)	0.96 (0.85, 1.07)
Q3	1.08 (0.98, 1.18)	1.12 (0.98, 1.27)	1.11 (1.01, 1.23)	1.05 (0.93, 1.18)
Q4	1.09 (0.99, 1.20)	1.09 (0.95, 1.24)	1.11 (1.00, 1.22)	1.03 (0.91, 1.17)
Q5(Highest)	1.03 (0.93, 1.14)	1.17 (1.01, 1.35)	1.16 (1.05, 1.29)	1.00 (0.88, 1.15)
**LAN**		**WHITE**		
Q1(Lowest)	Ref	Ref	Ref	Ref
Q2	1.05 (0.96, 1.16)	1.12 (0.97, 1.30)	1.02 (0.92, 1.13)	1.01 (0.89, 1.14)
Q3	1.02 (0.91, 1.14)	1.09 (0.92, 1.30)	0.98 (0.87, 1.11)	1.10 (0.96, 1.27)
Q4	0.98 (0.86, 1.12)	0.95 (0.77, 1.18)	1.01 (0.88, 1.17)	1.07 (0.90, 1.26)
Q5(Highest)	0.92 (0.80, 1.07)	1.23 (0.98, 1.54)	1.07 (0.91, 1.25)	1.28 (1.07, 1.55)
**LAN**		**TOTAL**		
Q1(Lowest)	Ref	Ref	Ref	Ref
Q2	1.04 (0.98, 1.11)	1.14 (1.03, 1.25)	1.06 (0.99, 1.13)	0.99 (0.91, 1.07)
Q3	1.05 (0.98, 1.13)	1.11 (1.00, 1.23)	1.07 (0.99, 1.15)	1.09 (0.99, 1.19)
Q4	1.04 (0.97, 1.12)	1.06 (0.95, 1.18)	1.06 (0.98, 1.15)	1.07 (0.97, 1.17)
Q5(Highest)	0.98 (0.90, 1.06)	1.16 (1.04, 1.31)	1.11 (1.02, 1.21)	1.08 (0.97, 1.20)

Models were adjusted for race (total sample alone) age, sex, education, household income, metropolitan status of residence, and deprivation index. Ref=Reference category; OR = Odds Ratio

Supplementary Table 2: Multinomial regression analysis of neighborhood noise levels and sleep durations at baseline and follow-up

	Baseline		Follow-up	
Characteristic	Short sleep	Long sleep	Short sleep	Long sleep
	OR	OR	OR	OR
**NOISE**		**BLACK**		
Q1(Lowest)	Ref	Ref	Ref	Ref
Q2	1.07 (0.98, 1.17)	1.12 (0.99, 1.26)	0.95 (0.87, 1.04)	0.96 (0.85, 1.07)
Q3	1.10 (1.01, 1.20)	1.13 (1.00, 1.28)	1.02 (0.94, 1.12)	1.00 (0.89, 1.12)
Q4	1.17 (1.07, 1.28)	1.11 (0.98, 1.26)	1.10 (1.00, 1.21)	1.02 (0.91, 1.15)
Q5(Highest)	1.11 (1.01, 1.22)	1.17 (1.03, 1.33)	1.16 (1.06, 1.28)	1.00 (0.89, 1.13)
**NOISE**		**WHITE**		
Q1(Lowest)	Ref	Ref	Ref	Ref
Q2	1.02 (0.93, 1.12)	1.00 (0.86, 1.16)	1.02 (0.92, 1.12)	1.10 (0.97, 1.24)
Q3	0.98 (0.88, 1.08)	1.11 (0.94, 1.30)	0.94 (0.84, 1.05)	1.05 (0.92, 1.20)
Q4	1.07 (0.95, 1.20)	1.03 (0.86, 1.24)	1.01 (0.89, 1.14)	1.04 (0.90, 1.21)
Q5(Highest)	1.02 (0.89, 1.17)	1.13 (0.92, 1.39)	1.01 (0.87, 1.17)	1.18 (1.00, 1.40)
**NOISE**		**TOTAL**		
Q1(Lowest)	Ref	Ref	Ref	Ref
Q2	1.04 (0.98, 1.11)	1.07 (0.98, 1.18)	0.98 (0.91, 1.04)	1.03 (0.95, 1.11)
Q3	1.05 (0.98, 1.12)	1.12 (1.01, 1.23)	0.99 (0.93, 1.06)	1.03 (0.95, 1.13)
Q4	1.13 (1.05, 1.21)	1.08 (0.98, 1.20)	1.08 (1.00, 1.16)	1.06 (0.96, 1.15)
Q5(Highest)	1.05 (0.98, 1.14)	1.14 (1.03, 1.27)	1.11 (1.03, 1.20)	1.06 (0.97, 1.17)

Models were adjusted for race (total sample alone) age, sex, education, household income, metropolitan status of residence, and deprivation index. Ref=Reference category; OR = Odds Ratio
